# Results of A Study On The Relationship Between Depression and Treatment Complications in Cancer Patients

**DOI:** 10.1192/j.eurpsy.2026.11404

**Published:** 2026-07-31

**Authors:** B. Purev, D. Dulamdorj, P. Sarantuya, M. Amartuvshin, B. Altankhuyag, K. Ganchuluun, K.-U. Mijgee

**Affiliations:** 1 Department of Medical Science, Etugen University; 2 School of Education, Department of Psychology, Mongolian National University of Education; 3 Department of Medical Science, Etugen University; 4 Manager of the Mental Health Service Center, Mongolian National University of Education, Ulaanbaatar, Mongolia

## Abstract

**Introduction:**

The global average incidence of cancer is 9.3 per 100,000 population, whereas in our country this figure is 93.7 — about 10 times higher. As cancer is diagnosed at more advanced stages, pain, treatment complications, financial burden, changes in employment, and family relationships become factors leading to depression, anxiety, and fear in patients. Depression is the most common psychological symptom among cancer patients. According to studies conducted in the United States, anxiety occurs in 18–25% of cancer patients, fear of recurrence after treatment in 80%, post-traumatic stress disorder in 5–30%, cancer-related stressors (family, economic, health status, bodily changes) in 30–40%, and depression in 10–30% of patients.

**Objectives:**

To study the relationship between the level of depression and treatment complications in cancer patients

**Methods:**

The study was conducted using a single-point analytical research design. Data were collected from 95 cancer patients receiving care at a family health center between February 10, 2025, and March 20, 2025, using the PHQ-9, 15 General Information Questionnaires, and a total of 24 questionnaires issued by the WHO for primary health care providers

**Results:**

Of the study participants, 26.3% (25) were male and 73.7% (70) were female. Among all participants, 39 (41.1%) were not depressed, 25 (26.3%) had self-limiting depression, 15 (15.8%) had moderate depression, 3 (3.2%) had moderate to severe depression, and 13 (13.7%) had severe depression.

The relationship between depression and treatment complications was assessed using Pearson’s correlation coefficient, and no significant correlation was found (r = –0.129).

**Conclusions:**

The majority of cancer patients are depressed. Severe depression can make it difficult to engage in simple social activities and may even lead to suicide; therefore, psychiatric evaluation, diagnosis, and treatment are necessary.

**Disclosure of Interest:**

None Declared

